# Assessing the Utilization of Electronic Consultations in Genetics: Seven-Year Retrospective Study

**DOI:** 10.2196/63028

**Published:** 2025-04-30

**Authors:** Sawona Biswas, Joyce So, Robert Wallerstein, Ralph Gonzales, Delphine Tout, Lisa DeAngelis, Aleksandar Rajkovic

**Affiliations:** 1 Department of Pathology, University of California San Francisco, 2340 Sutter Street, San Francisco, CA, 94115, United States, 1 415-514-7648; 2 Department of Pediatrics, University of California, San Francisco, San Francisco, CA, United States; 3 Department of Medicine, University of California, San Francisco, San Francisco, CA, United States; 4 Division of Nephrology, University of California, San Francisco, San Francisco, CA, United States; 5 Ambulatory Patient Care Access, UCSF Health, San Francisco, CA, United States; 6 Department of Obstetrics, Gynecology & Reproductive Sciences, University of California, San Francisco, CA, United States

**Keywords:** genomic, e-Consult, genetic, utility, retrospective, assessment, effectiveness, electronic consultation, healthcare providers, genetic experts, university, consultations, e-Consult frameworks, accessibility, genetic testing, patient care

## Abstract

**Background:**

Patient and health care provider access to genetic subspecialists is challenging owing to limited number of genetics experts across the United States. The University of California San Francisco (UCSF) Genetics electronic consultation (e-Consult) service was implemented along with the usual referral pathway to improve access to timely genetic expertise through robust asynchronous provider-to-provider communication.

**Objectives:**

This study examined the impact of the UCSF Genetics e-Consult service on patient access to genetics expertise.

**Methods:**

A retrospective chart review of 622 e-Consult requests was conducted. Data pertinent to e-Consult completion rates, provider response times, consultation content, and adherence to geneticist recommendations were abstracted.

**Results:**

From October 2016 to March 2024, the UCSF Genetics e-Consult service received a total of 622 consultation orders, with yearly volumes increasing from 34 in 2017 to 144 in 2023. A total of 360/622 (57.8%) consultations were completed, of which 197/360 (54.6%) were resolved without requiring a specialty care visit. Of the 262/622 (42.1%) e-Consult orders declined by the geneticist reviewer, 184/262 (70.2%) were scheduled for a synchronous genetics visit due to case complexity precluding an appropriate e-Consult response and 29.8% (78/262) were recommended to be referred to a different and more appropriate specialty. Geneticists responded to 83.9% (522/622) of e-Consults within 3 days, with most spending between 5 and 20 minutes on their e-Consult response. Nearly half of the genetics e-Consult requests (69/144; 47.9%) came from primary care providers and pediatricians. Among the 144 e-Consult requests in 2023, 50.6% (73/144) were about diagnostic queries, 17% (25/144) were on symptom management, and 11% (16/144) were about test interpretation. Provider adherence to geneticists’ recommendations was observed in 84% (116/144) of cases.

**Conclusions:**

The UCSF Genetics e-Consult service has demonstrated efficiency in providing timely genetic consultations, with a high rate of provider adherence to recommendations. These findings support the potential of e-Consult frameworks as a viable strategy for enhancing access to genetic health care services.

## Introduction

The field of genetics and genomics has experienced significant advancements in recent years, leading to increased integration of genetic testing and considerations into various medical disciplines. However, the number of genetic subspecialists remains limited, resulting in challenges for patients and health care providers seeking timely access to genetics expertise [1,2]. The shortage of genetic specialists, coupled with the growing demand for their services, has led to prolonged waiting times for appointments and potential delays in patient care [3].

As genetic testing is increasingly incorporated into the diagnostic and treatment processes across medical specialties, there is a pressing need to improve communication and collaboration between genetic specialists and a wide range of health care providers [4]. Primary care clinicians, specialists in other disciplines, and allied health professionals often require guidance and support when interpreting genetic test results, understanding their implications, and supporting patient informed decisions regarding their management [5].

To address these challenges, health care systems have explored innovative solutions, such as electronic consultations (e-Consults), to facilitate prompt access to specialist expertise [6]. e-Consults have emerged as a promising approach to bridge the gap between the limited number of genetic specialists and the growing demand for their service. Within genetics and genomics programs, e-Consult initiatives have demonstrated their effectiveness in addressing basic questions without the need for an in-person visit and providing educational opportunities for generalists to enhance their knowledge and skills in genetics care [7-10]. As genetics becomes increasingly integrated into mainstream medical practice, e-Consults serve as a valuable tool to foster collaboration, ensure knowledge sharing, and provide high-quality, genetics-informed care to patients.

The e-Consult service at the University of California San Francisco (UCSF) started in 2012 [11]. Initially, the program was grant funded and then financially supported by the health system. By 2015, the UCSF entered into agreements with three payers to receive reimbursement for specialist e-Consults, and by 2020, the Centers for Medicare and Medicaid Services (CMS) and other payers started providing reimbursement for e-Consults that are completed and not declined. The introduction of Genetics e-Consults occurred in June 2017, positioning the UCSF as an early adopter in the United States. Herein, we describe the establishment of our Genetics e-Consult service for providers within our institution and its utilization.

## Methods

### Study Structure

This study analyzed e-Consults submitted through the UCSF Health’s electronic medical record system. Providers across multiple specialties, including primary care, pediatrics, and oncology, initiated consultations. The analysis included all Genetics e-Consults from October 2016 to March 2024, capturing utilization trends and consultation characteristics.

### Process of e-Consult Services

Ordering providers within UCSF Health can initiate an e-Consult request through our hospital’s electronic medical record (Epic Hyperspace 2023; developed by Epic Systems Corporation, Verona, WI, USA). Providers can choose from the Adult Genetics, Pediatrics Genetics, or Cancer Risk Genetics service. After selecting the service, the ordering providers will be prompted to choose one of the four diagnosis-specific SmartText templates: (1) Query genetic condition or syndrome, (2) Known genetic condition or syndrome, (3) Family history of genetic condition, or (4) Other. Each template will guide the provider through different open-ended questions that capture the reason for the e-Consult, relevant test results, family history information, and any other pertinent information. After submission, the e-Consult request is routed to the designated pool and the e-Consultant on service reviews the details and responds.

The Genetics e-Consultants are a group of board-certified geneticists practicing in the Pediatric or Adult Genetics service at the UCSF with more than 5 years of experience. The e-Consultant after accepting the referral summarizes or refines the consultative questions, offers an individualized recommendation along with supporting evidence or current best practices, and outlines a contingency plan (including guidelines for when to refer the patient for an in-person or telehealth specialty visit). The standard response time for an e-Consult is set at 3 business days. In cases where the clinical complexity exceeds the scope of an e-Consult, the e-Consultant may opt to recommend a traditional new patient appointment. The e-Consultant will cc: their specialty staff on the e-Consult response who then convert the e-Consult order to a referral order for routing to the appropriate scheduling team (upon submittal, the requester indicates okay to convert to referral or not okay to convert). Alternatively, if the consultation falls outside the consultant’s area of expertise and is better suited for another specialty, the e-Consultant will decline the e-Consult with that recommendation. The e-Consultant self-reports the time spent in the electronic medical record before marking the consultation as complete. The median of these recorded times was then calculated. Within our department, a dedicated team of geneticists is responsible for addressing e-Consults. Once the e-Consult is completed or declined, it is returned to the referring provider’s Epic In Basket (Epic Systems Corporation) an integrated electronic messaging and task management system within the Epic electronic health record, with either the clinical recommendations or reason for decline. A short 8-question provider survey was conducted to assess the utilization, satisfaction, and impact of the e-Consult service.

### Retrospective Chart Review

A retrospective chart review was conducted to explore the details of e-Consults ordered from October 2016 to March 2024. The review did not require institutional review board approval at the UCSF. Our group characterized the ordering providers, departments, referral groups, descriptions of the consult, primary referral diagnosis, consult status, response times, e-Consult recommendations, number of related referrals to Genetics, and tests performed. Data were collected from EPIC reports and individual chart review, and descriptive statistical analysis was completed using R software (version 4.4.3; R Foundation for Statistical Computing). and Microsoft XML. Statistical reporting included all e-Consult activity originating from an order. Per UCSF Health e-Consult EPIC workflow, all e-Consult orders result in a completed and closed encounter for reporting, whether completed with a clinical recommendation or declined for the reason indicated.

### Ethical Considerations

This retrospective quality control and quality improvement study involved a chart review of deidentified data from the electronic medical records at the UCSF. The study was conducted solely for the purpose of evaluating and improving the existing Genetics e-Consult service within the institution. As this project did not involve primary data collection and was intended for internal quality improvement with no impact on patient care decisions or patient rights, it did not constitute human subjects research under the purview of the Department of Health and Human Services. Accordingly, this study did not require Institutional Review Board review or approval per UCSF’s internal policies and federal regulations. All study data were deidentified and securely stored within UCSF’s electronic health records system. No financial compensation was provided to study participants, as the research involved retrospective data analysis. No images or materials include identifiable patient information. This not a funded project. All authors are UCSF employees.

## Results

**Figure 1.**
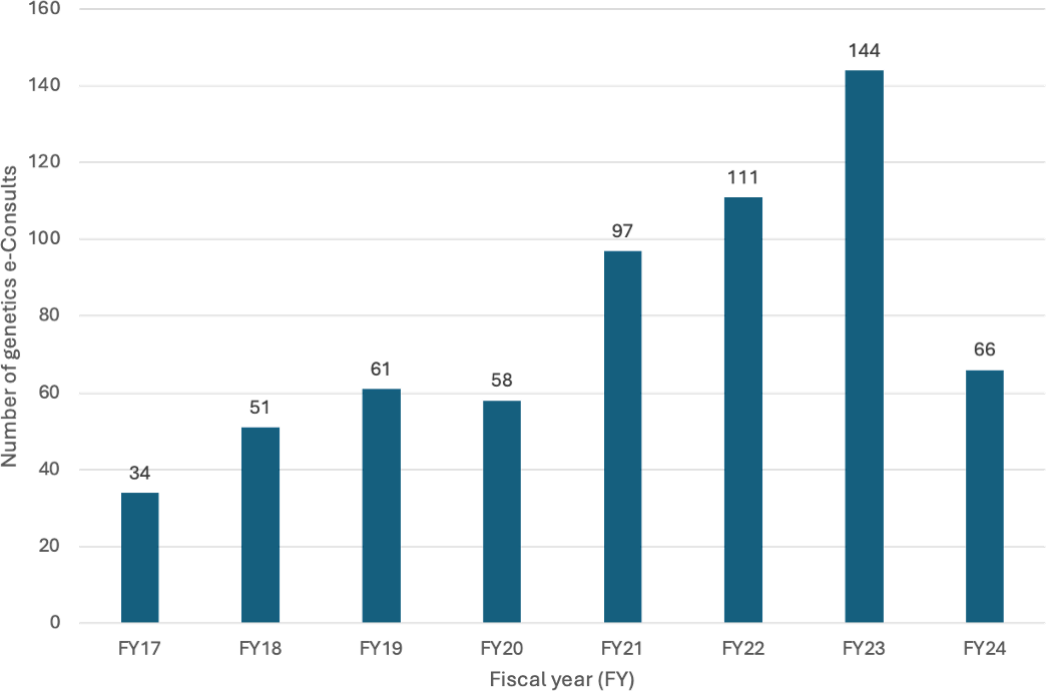
Total number of e-Consults received by genetics (Adult Genetics, Pediatrics Genetics, or Cancer Risk Genetics) at the University of California, San Francisco from Oct 01, 2016 till 01 March, 2024.

From October 2016 to March 2024, the UCSF Genetics e-Consult service received a total of 622 consultation orders, with yearly volumes increasing from 34 in fiscal year (FY) 2017 to 144 in FY 2023 (Figure 1).

**Figure 2.**
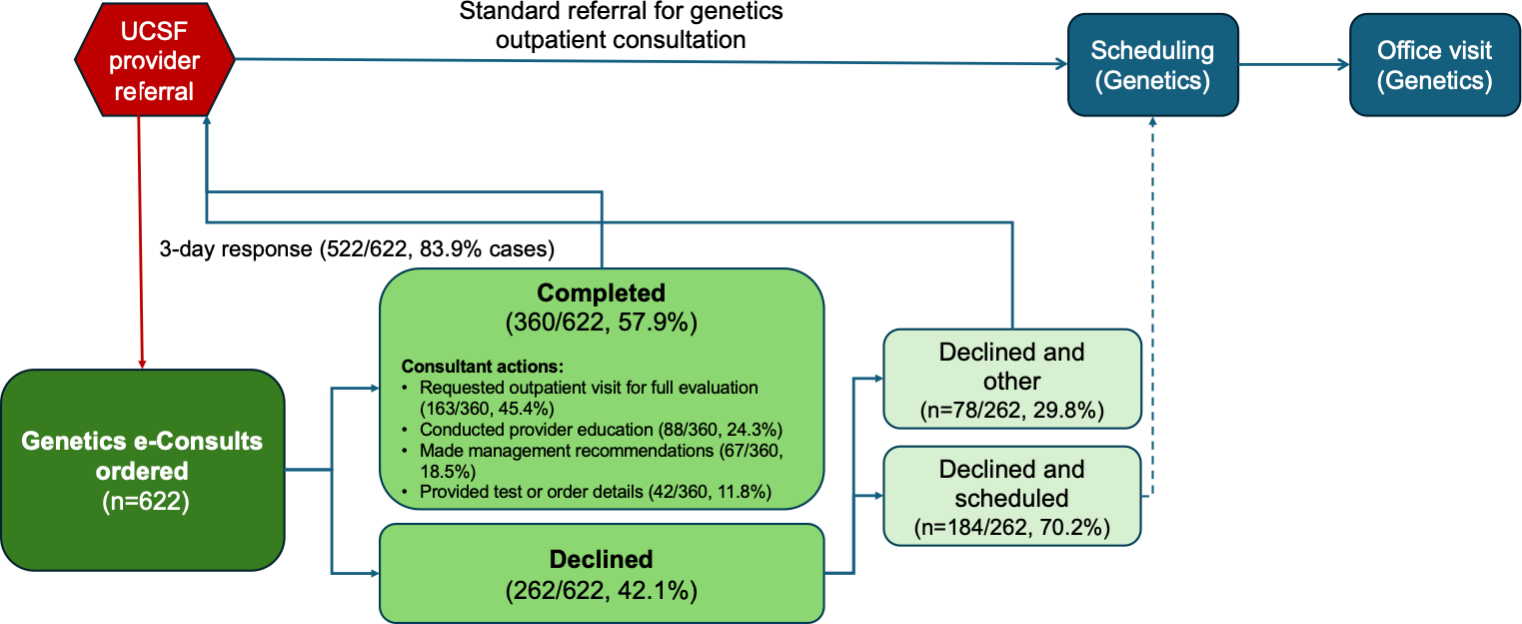
Flowchart and breakdown of e-Consults received and responded 2017‐2024. UCSF: University of California, San Francisco

Of the 622 Genetics e-Consults, 360/622 (57.8%) were completed, while 262/622 (42.1%) were either declined or deferred to another specialty (Figure 2). Responses were provided on the same day for 263/622 consults (42.3%), within 3 days for 522/622 consults (83.9%), and within 7 days for 562/622 consults (90.4%; Multimedia Appendix 1).

**Figure 3.**
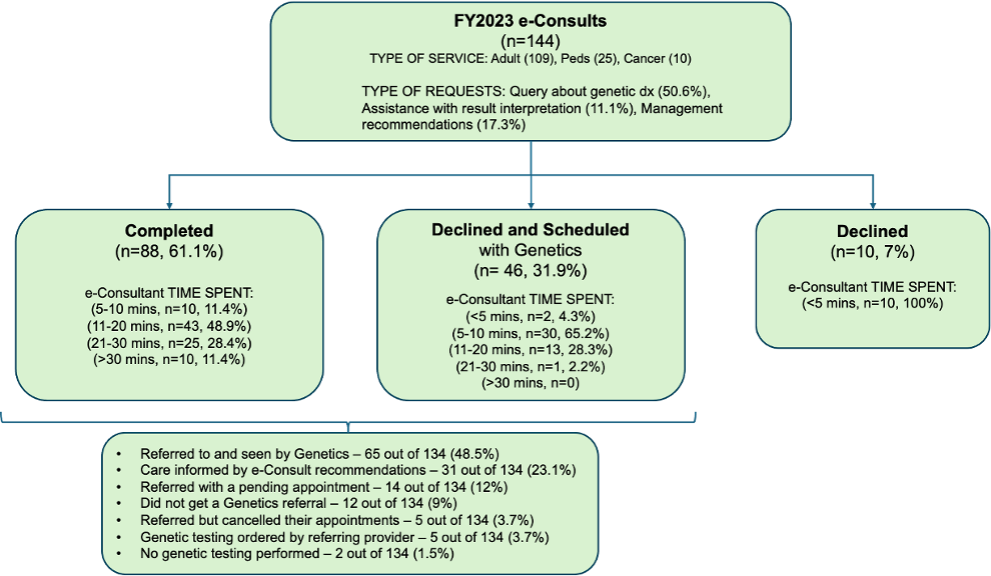
Breakdown of e-Consults received in the fiscal year (FY) 2023.

A comprehensive case-level analysis was conducted for the 144 e-Consults ordered during the FY 2023 (June 2022 to July 2023), as this was the first year in which the e-Consult service was fully integrated, with standardized workflows, defined practices, and distinct divisions for Adult, Pediatric, and Cancer Genetics. Prior years lacked the necessary structure and documentation for a comparable analysis. Adult Genetics received the most consults (109/144, 75.7%), followed by Pediatric Genetics (25/144, 17.4%) and Adult Cancer Risk Genetics (10/144, 6.9%). A total of 88/144 (61.1%) of these e-Consults were completed with detailed responses, 46/144 (31.9%) were declined and converted to in-person Genetics appointments, and 10/144 (6.9%) were declined for other reasons such as insufficient information or referral to another specialty (Figure 3).

Nearly half (69/144, 47.9%) of the FY 2023 e-Consults originated from general practices such as primary care, general medicine, and women’s health. The median time to answering an e-Consult was 15 (IQR 10‐25) minutes. e-Consultants spent less than 5 minutes in all the declined cases and an average of 11‐20 minutes on completed cases and mostly 5‐10 minutes on cases that were declined and scheduled with Genetics (Figure 3). The most common types of e-Consult requests were queries about genetic diagnoses (73/144, 50.6%), assistance with test interpretation (16/144, 11.1%), and management recommendations for patients with known genetic diagnoses (13/144, 9%) or unknown genetic diagnoses (12/144, 8.3%; Multimedia Appendix 1). e-Consultants provided a range of services, with the most frequent being scheduling patients for outpatient visits (61/144, 42.3%), providing genetic counseling and management recommendations (35/144, 24.3%), making management recommendations alone (27/144, 18.5%), and offering guidance on genetic testing (17/144, 11.8%) and recommended genetics referrals (5/144, 3.4%; Multimedia Appendix 1). A chart review of 134 e-Consults (88 completed and 46 scheduled) revealed that 65/134 patients (48.5%) were referred to and seen by Genetics, 14/134 (12%) were referred with a pending appointment, 5/134 (3.7%) were referred but canceled their appointments, 31/134 (23.1%) had their care informed by the e-Consult recommendations, 5/134 (3.7%) had genetic testing ordered by their referring provider, 12/134 (9%) did not get a Genetics referral, and 2/134 (1.5%) did not have any genetic testing performed (Figure 3).

## Discussion

Our study demonstrates the successful implementation and growth of an e-Consult service that connects general providers and specialists with genetics experts at a large academic medical center. The UCSF Genetics e-Consult service experienced a steady increase in volume from 34 consults in FY 2017 to 144 in FY 2023 (Figure 1), highlighting the growing demand for accessible genetics expertise.

The e-Consult service provided an efficient means to triage genetics questions, with 57.8% (360/622) of the submitted consults completed and 42.1% (262/622) declined, primarily to schedule in-person evaluations instead (184/262, 70.2% of declined cases). The average turnaround time of 3 days demonstrates the responsiveness of the service in addressing clinical questions.

An analysis of 144 e-Consults from FY 2023 revealed that Adult Genetics handled the majority 109/144 (75.7%), followed by Pediatric Genetics 25/144 (17.4%) and Adult Cancer Risk Genetics 10/144 (6.9%). While 88/144 (61.1%) of these consults were completed electronically, 46/144 (31.9%) were declined and converted to in-person visits (Figure 3), suggesting that e-Consults can help determine when face-to-face evaluations are needed. Nearly half (69/144, 47.9%) of FY 2023 e-Consults came from generalists in primary care, general medicine, and women’s health, indicating broad adoption across nongenetics specialties. e-Consultants provided prompt responses, spending 20 minutes or less on 75% of the cases (Figure 3).

The most common reasons for e-Consults were questions about genetic diagnoses (73/144, 50.6%), test interpretations (16/144, 11.1%), and patient management (Multimedia Appendix 1). e-Consultants frequently recommended clinic visits (61/144, 42.3%), provided counseling and management advice (35/144, 24.3%), and offered guidance on genetic testing (17/144, 11.8%; ). Referral outcomes data demonstrated that e-Consults resulted in 62% (84/134) of patients being seen by or scheduled with appointments at Genetics, while for 20% of patients (31/134), e-Consult advice influenced their care without necessitating a formal referral to Genetics.

These findings align with and expand upon the limited existing literature on Genetics e-Consult programs. Similar to studies by Bhola et al [9] and Folkerts et al [10], we observed efficient turnaround times, a wide range of clinical topics addressed, and high rates of guideline-concordant care when e-Consults provided actionable recommendations. Our higher e-Consult volume likely reflects the longer duration and wider scope of our program.

The high proportion of e-Consults completed electronically (360/622, 57.9%) highlights the potential for e-Consults to optimize genetics care delivery by addressing straightforward questions, triaging complex cases for in-person evaluation, and guiding appropriate previsit workups. The 84% adherence rate among providers to e-Consult recommendations underscores the educational impact of specialist guidance.

The limitations of our study include the single-center design, lack of long-term outcome data, and absence of referring provider and patient perspectives. Future research should compare e-Consult programs across diverse health care settings; incorporate feedback from key stakeholders; and evaluate the impact on care access, quality, and costs.

In conclusion, the UCSF Genetics e-Consult service demonstrates a scalable and efficient model for expanding access to genetics expertise. By providing timely, individualized guidance to nongenetics providers, e-Consults can help optimize resource utilization, provider education, and patient care as genetics becomes increasingly integrated into routine clinical practice.

## Supplementary material

10.2196/63028 Multimedia Appendix 1 Supplementary tables.

## Abbreviations

CMS: Centers for Medicare and Medicaid Services
e-Consult: electronic consultation
FY: fiscal year
UCSF: University of California San Francisco
