# Supplementary material for: Assessing the Utilization of Electronic Consultations in Genetics: Seven-Year Retrospective Study
Source: JMIR Form Res. 2025 Apr 30;9:e63028. doi: 10.2196/63028 (PMC12058035; doi:10.2196/63028)
Supplement: Multimedia Appendix 1 [file formative-v9-e63028-s001.docx]

TABLE 1:

|  |  | **FY 2017** | **FY 2018** | **FY 2019** | **FY 2020** | **FY 2021** | **FY 2022** | **FY 2023** | **FY 2024*** | **Total To Date** |
| --- | --- | --- | --- | --- | --- | --- | --- | --- | --- | --- |
| Specialty | Start Date | Total eConsult Orders | Total eConsult Orders | Total eConsult Orders | Total eConsult Orders | Total eConsult Orders | Total eConsult Orders | Total eConsult Orders | Total eConsult Orders | Total eConsult Orders |
| Adult Cancer  Risk Genetics | 3/1/2023 |  |  |  |  |  |  | 10 | 9 | 19 |
| Adult Genetics | 7/1/2020 |  |  |  |  | 88 | 100 | 109 | 52 | 349 |
| Pediatric Genetics | 4/1/2020 |  |  |  |  | 9 | 11 | 25 | 5 | 50 |
| Pediatric and  Adult Genetics | 10/1/2016 | 34 | 51 | 61 | 58 |  |  |  |  | 204 |
| **TOTAL** |  | **34** | **51** | **61** | **58** | **97** | **111** | **144** | **66** | **622** |

Table 1: Total number of e-Consults received by Genetics at UCSF from 10/01/2016 till 03/01/2024.

TABLE 2:

| **Genetics e-Consults - Decline Reasons** | **Volume of Declines** | **% Distribution of Decline Reasons** |
| --- | --- | --- |
| Decline and Schedule | 184 | 70.2% |
| Decline, PCP Preference not to schedule | 1 | 0.4% |
| Decline, Established patient & problem | 3 | 1.1% |
| Decline, Insufficient Info | 3 | 1.1% |
| Decline, Other | 71 | 27.1% |
| **Total** | **262** | **100.0%** |

Table 2: Details of the total 262 e-consults declined and reasons.

TABLE 3:

| **Response Time (**Goal = 3 BUSINESS days**)** | **% Answered Same Day** | **% Answered** **within 3 Days** | **%Answered**  **within 7 Days** |
| --- | --- | --- | --- |
| **Genetics** | 42.3% | 83.9% | 90.4% |
| **UCSF Program - All Specialties** | 37.6% | 87.9% | 95.9% |

Table 3: Percentage response times of the total 622 e-consults orders answered within the same day, three business days and seven business days.

TABLE 4:

| **Distribution of e-Consultant Time Spent** | **%** |
| --- | --- |
| Under 5 minutes | 7.9% |
| 5-10 minutes | 28.0% |
| 11-20 minutes | 39.1% |
| 21-30 minutes | 17.8% |
| 31 minutes or greater | 7.1% |
| **Total** | 100.0% |

Table 4: Distribution of e-Consultant time spent on each consult for all 622 e-Consults.

TABLE 5:

| **Category of e-Consult request** | **Count** | % |
| --- | --- | --- |
| Query genetic diagnosis | 73 | 50.7% |
| Help with test interpretation | 16 | 11.1% |
| Help with management of patient(s) with known genetic diagnosis | 13 | 9.0% |
| Help with management of patient(s) without known genetic diagnosis | 12 | 8.3% |
| Help with identifying the right test order, laboratory, or how to order | 12 | 8.3% |
| Help with referral appropriateness | 7 | 4.9% |
| Family history of a genetic condition | 7 | 4.9% |
| Prenatal/Preconception counseling | 4 | 2.7% |
| **Grand Total** | **144** |  |

Table 5: Details of the types of e-Consults requested in Fiscal Year 2023 (count and percentages).

TABLE 6:

|  | **Count** | **Total count per group** |
| --- | --- | --- |
| Schedule for outpatient visit | 54 |  |
| Schedule for outpatient visit, and management recommendations made | 4 | 61(42.3%) |
| Schedule for outpatient visit, and interpreted test results | 1 |  |
| Schedule for outpatient visit, and provided test details with or without links | 2 |  |
| Conducted provider education, and management recommendations made | 15 |  |
| Conducted provider education, and recommended genetics referral | 7 | 35 (24.3%) |
| Conducted provider education, and recommended to test family members | 6 |  |
| Conducted provider education, and provided test details with or without links | 1 |  |
| Conducted provider education | 6 |  |
| Management recommendations made | 13 |  |
| Management recommendations made, and interpreted test results | 5 | 26 (18.0%) |
| Management recommendations made, and recommended genetics referral | 4 |  |
| Management recommendations made, provided test details with or without links | 2 |  |
| Management recommendations made, and recommended to test family members | 2 |  |
| Provided test details with or without links | 10 |  |
| Provided test details with or without links, and recommended genetics referral | 5 | 17 (11.8%) |
| Provided test details with or without links, and interpreted test results | 2 |  |
| Recommended genetics referral | 4 |  |
| Recommended genetics referral, and interpreted test results | 1 | 5 (3.4%) |
| **Grand Total** | **144** |  |

Table 6: Details of the types of action(s) or outcome(s) undertaken by the e-Consultant in fiscal year 2023 after the e-Consult is completed (count, total count per group, and percentages).
